# IL-2 Combined with IL-15 Enhanced the Expression of NKG2D Receptor on Patient Autologous NK Cells to Inhibit Wilms' Tumor via MAPK Signaling Pathway

**DOI:** 10.1155/2022/4544773

**Published:** 2022-09-30

**Authors:** Yanping Li, Yonglin Li, Bin Xiang, Xiaomao Tian, Qinlin Shi, Feng Liu, Tao Lin, Guanghui Wei

**Affiliations:** ^1^Ministry of Education Key Laboratory of Child Development and Disorders, Chongqing Key Laboratory of Pediatrics, Chongqing Key Laboratory of Children Urogenital Development and Tissue Engineering, China International Science and Technology Cooperation Base of Child Development and Critical Disorders, Pediatric Research Institute, Children's Hospital of Chongqing Medical University, Chongqing 400014, China; ^2^Department of Pediatric Urology Surgery, Children's Hospital of Chongqing Medical University, Chongqing 400014, China

## Abstract

**Objective:**

The dysfunction of immune surveillance, a hot spot in cancer research, could lead to the occurrence and development in multicancers. However, the potential mechanisms of immunity in Wilms' tumor (WT) remain unclear on Wilms' tumor (WT). In this study, we aim to investigate the immune cell in WT and explore the underlying treatment strategy.

**Method:**

We quantified stromal and immune scores by using ESTIMATE algorithm based on gene expression matrix of WT patients in TCGA and GEO databases. Different expression genes (DEGs) and functional enrichments were analyzed by R studio and DAVID tools. Flow cytometry, immunofluorescence staining, ELISA assay, and qRT-PCR were used for detecting the NK cells, cytotoxic cytokines (INF-*γ*, PRF, and GZMB), and NK cell receptor expression, respectively. WT patient autologous NK cells were stimulated by IL-2 and IL-15, and the cytotoxicity of NK cells against WT cell lines was detected by LDH assay. Western blot experiment was used for measuring the MAPK signaling pathway protein maker in NK cells.

**Results:**

ESTIMATE indicated that WT tissue had a lower immune score than adjacent kidney tissue. Meanwhile, the low immune score group was associated with poorly outcomes. DEG functional enrichment analysis showed that NK cell-mediated cytotoxicity was significantly different in low and high immune score groups. Although few of proportion of NK cells in WT patients were increased, most of that were significantly lower than normal children. Moreover, the proportion of NK cells and the expression level of INF-*γ*, PRF, and GZMB in WT tissue were lower than adjacent kidney tissue. Importantly, the NKG2D expression level of NK cells was significantly lower in WT tissue. Furthermore, in vitro, compared with uncultured NK cells, IL-2 and IL-15 could effectively enhance the cytotoxicity of NK cells on killing the WT cell lines. The FACS and WB results showed that the NKG2D and p-PI3K ratio PI3K, MEK1/2, and p-ERK1/2 ratio ERK1/2 were significantly increased in IL-2 and IL15 group compared with uncultured groups.

**Conclusion:**

The abnormal NK cell-mediated cytotoxicity may cause the occurrence of WT. Costimulation of WT patients autologous NK cells could effectively enhance the antitumor reaction which involved in activation of NKG2D-mediated MAPK signaling pathway.

## 1. Introduction

Wilms' tumor (WT), the most common malignant solid tumor of children, was often diagnosed before the age of 4 years [1]. Currently, the treatment strategy is multidisciplinary (surgery, chemotherapy, and radiotherapy) and individualized treatment which bring significant benefit on patients with WT [2]. However, there are still some challenges on treatment WT, that is, anaplasia, metastasis, recurrence, and insensitivity to chemotherapy and radiotherapy [3]. Meanwhile, a number of researchers have paid attention to the long-term subhealth caused by radiotherapy and chemotherapy. Therefore, to explore the new treatments is compelling.

Tumor immune therapy, a revolutionary treatment in the 21st century, has received widespread attention in both adults and children tumor. Once the balance of immune system is broken, the tumor cells escaped from immune surveillance, which led to tumor occurrence and development [4].

The tumor microenvironment (TME), a mixture that consists of tumor-infiltrating immune cells (TIICs), mesenchymal cells, endothelial cells, extracellular matrix molecules, and inflammatory mediators, is close relationship between the occurrence, growth, and metastasis of tumor and the internal and external environments of tumor cells which includes not only the structure, function, and metabolism of tumor tissue but also the internal (nuclear and cytoplasmic) environment of tumor cells themselves [5, 6].

Although some studies showed that WT microenvironment is immune engaged and may be susceptible to immunotherapy similar to other malignancies, few studies have systematic analysis of TIIC composition [7].

Recently, ESTIMATE algorithm [8] was applied to value stromal and immune microenvironment infiltration based on gene expression data which give an overall view of TME. Among the cells, infiltrating stromal and immune cells are the two key contributors to the TME, which significantly associated with the cancer biology [9].

Therefore, in this study, first, TCGA-WT and GEO datasets were used to explore the immune and stromal cells by ESTIMATE algorithm. According to the above results, differentially expressed genes (DEGs) of high and low immune cell infiltration were analyzed for enrichment pathway by DAVID tools. A key pathway, NK cell-mediated cytotoxicity, was significantly different in high and low immune cell score group. NK cells, as innate lymphoid cells, play an important role in the first line of defense toward transformed cells [10]. Furthermore, the count of NK cells in WT patient samples and cytotoxicity of NK cells were detected. The previous reported that cytokines play a major role in the regulation of the cellular responses between tumors and the immune system [11]. Here, we demonstrated that cytokine (IL-2 and IL-15) could enhance the cytotoxicity of WT patient autologous NK cells against WT cells line. The specific mechanisms may be involved in enhancing NKG2D-mediated MAPK signaling.

## 2. Material and Method

### 2.1. Study Sample

RNA-sequence and clinic data for WT patients which contained 124 WT samples and 6 normal kidney samples were downloaded from the TCGA-Target-WT data portal (https://tcga-data.nci.nih.gov/tcga/) using package “TCGAbiolinks” in R. GEO datasets (GSE66405, GSE73209, GSE53224, GSE31403, and GSE40435) were download from GEO NCBI.

Our study included thirty patients with WT stages I-V which diagnosed by the postoperation pathology, according to National Wilms Tumor Study- (NWTS-) 5 standard. Peripheral blood and paired tumor adjacent nontumor tissue samples were collected from preoperation and postoperation. Patients were not included if they had undergone any cancer treatment before this study. 30 cases of healthy children were used as controls. The study was approved by the Research Ethics Committee of Children Hospital of Chongqing Medical University, and all study participants signed an informed consent form.

### 2.2. NK Cell Count

The previous study showed that the NK cells were analyzed with the help of a marker on flow cytometry [12]. In this study, 200 *μ*L heparinized whole blood samples were surface-stained with 5 *μ*L FITC anti-human CD3 (BD Biosciences, 561806, USA), APC anti-human CD16 (Zen Biosciences, 790365, CN), PE anti-human CD56 (BD Biosciences, 561903, USA), and isotype control for 30 min at room temperature, respectively. The red cells were then lysed with red cell lyse buffer (BD, USA) for 30 min at room temperature and washed twice with 500 *μ*L phosphate buffer solution (PBS) via centrifugation. The resultant white cell pellets were resuspended and analyzed with a FACSCalibur flow cytometer (Becton Dickinson, San Jose, CA, USA). Lymphocytes were gated, and NK cell population was analyzed for the immunophenotypic features as CD3^−^CD16^+^CD56^+^ NK cells.

### 2.3. Immunofluorescence Staining

WT tissues and adjacent tissues (atrophy of the kidney tissue adjacent to the tumor) were harvested from patients who underwent partial or radical nephrectomy. Tumor tissues and adjacent tissues were fixed by 4% paraformaldehyde, dehydrated, paraffin embedded, and sectionalized. The tissue sections were dewaxed and hydrated with antigen repair using citrate (pH = 6.0). After being blocked by 0.5% BSA for 1 h, the tissue sections were incubated with primary antibodies, rabbit anti-human CD56 antibody (1 : 200, Proteintech, 14255-1-AP, USA) and mouse anti-human CD16 antibody (1 : 200, Proteintech, 66779-1-Ig, USA), at 4°C overnight and then hatched with secondary antibodies for 1 h. The nuclei were counterstained with Hoechst 33342 (Beyotime, C1022, China) with lucifugal for 30 min at room temperature. The tissue sections were sealed using the antifluorescence quenching agent and performed using a 90i fluorescence microscope (Nikon, Japan).

### 2.4. ELISA

The tissue protein levels of INF-*γ*, PRF1, and GzmB were measured by ELISA according to the ELISA kit (human IFN-*γ*, PRF1, GzmB ELISA Kit, Elabscience, China) instructions. Total proteins were isolated using precooling PBS. Total proteins were diluted 1 : 1 with sample dilution or nondilution total proteins. A volume of 100 *μ*L sample was added to the ELISA plate and incubated for 90 minutes at 37°C. Discard liquid and dry without washing. Biotinylated antibody working fluid (concentrated biotinylated detection Ab: biotinylated detection Ab: diluent = 1 : 100) was added and incubated for 60 minutes at 37°C. After washing three times, the conjugate (concentrated HRP conjugate: HRP conjugate diluent = 1 : 100) was added and incubated for 30 minutes at 37°C. After washing the plate five times, the substrate reagent (TMB) was added and incubated for 15 minutes at 37°C with lucifugal. The reaction was terminated by the termination solution. The optical densities of each well were measured with a microplate reader (Bioteck, USA) at a 450 nm wavelength. All samples were measured in duplicate.

### 2.5. Real-Time Reverse Transcription Polymerase Chain Reaction (qRT-PCR) Analyzes the NK Cell Receptors

Total RNA from tumor tissues and adjacent tissues was extracted using a TRIzol (Sigma, T9424, Germany) on the basis of the manufacturer's instruction. The RNA concentration and purity were measured by a NanoDrop 2000 spectrophotometer (Thermo Fisher Scientific). mRNA was reversely transcribed into cDNA using RT Master Mix for qPCR (gDNA digester plus) kit (MedChemExpress, HY-K0511, USA). Primers for NKG2D (KLRK1), D224 (GGT1), LAG3, IL-15RA, and GAPDH were shown in the table below. qRT-PCR was proceeded using SYBR Green qPCR Master Mix (No ROX) kit (MedChemExpress, HY-K0523, USA) with an ABI ViiATM7 real-time PCR system (Applied Biosystems, USA). The total reaction capacity was 10 *μ*L, including 1 *μ*L cDNA sample, 0.4 *μ*L primer, SYBR Green Master Mix (2x) (No ROX) 5 *μ*L, and DEPC H_2_O 3.6 *μ*L. The PCR program was initiated as an incipient step of 95°C for 5 min, followed by 40 cycles, denatured at 95°C for 30 s, and extended by 30 s at 60°C. The expression levels of mRNA were computed using the 2^-*ΔΔ*t^ means and standardized to GAPDH.

### 2.6. NK Cell Isolation and Expansion from PBMCs and NK Cell Activity Assay

Peripheral blood mononuclear cells (PBMCs) were isolated from WT patients with the use of Ficoll. PBMCs, containing NK cells, were in six plates at density of 3 × 10^5^ cells per well in 2 mL of RPMI-1640 medium supplemented with 10% FBS, 1,000 U/mL IL-2, 10 ng/mL IL-15, 20 ng/mL penicillin, and 100 mg/mL streptomycin (Gibco, NY, USA) every two days without removal of preexisting culture medium to maintain cellular concentration at 1‐2 × 10^6^ cells/mL for 7 days. The cells were observed under microscope and collected for flow cytometric analysis or for use in subsequent experiments. For NKG2D-NK cell analysis, before and after culture PBMCs, respectively, were surface stained with FITC anti-human CD3, PE anti-human CD56, and PerCP/Cyanine5.5 anti-human CD314 (NKG2D, BioLegend, 320817, USA) for 30 min at 4°C and washed twice with 500 *μ*L PBS via centrifugation. The expression level of NK cell activation receptor NKG2D was detected by FACS. For NK cells isolated, the NK cells were surface stained with FITC anti-human CD3 and PE anti-human CD56. The CD3^−^CD56^+^ cells were isolated by FACS. Purity NK cells were collected in RPMI-1640 medium (contain 10% FBS, 1,000 U/mL IL-2, 10 ng/mL IL-15, 20 ng/mL penicillin, and 100 mg/mL streptomycin) and used for subsequent experiments.

### 2.7. LDH Release Assay

NK cell cytotoxicity against tumor cells was analyzed using a lactate dehydrogenase (LDH) release assay. G401 and SK-NEP-1 cells (5 × 10^3^) were plated, and on the next day, NK cells were added at various ratios (1 : 1, 1 : 5, 1 : 10, and 1 : 20, target cells: effector cells) (all sample in triplicate). After 4 h of coculture, an aliquot of 50 *μ*L media was used in LDH cytotoxic assay using the LDH cytotoxic assay kit. The experimental release was corrected by subtraction of the spontaneous release of effector cells at corresponding dilutions. %cytotoxicity = (experimental value − effector cell spontaneous control − target cell spontaneous control) / (target cell maximum control − target cell spontaneous control) × 100.

### 2.8. Cytotoxic Cytokine Assay by a Flow Cytometry

NK cells were collected and resuspended on cold PBS including 2% FBS. After that, the suspension was added to a 6-well plate and staining by APC anti-human CD107a (LAMP-1) (BioLegend, 328619, USA) and incubated for 1 hour at 4°C in the dark. Golgiblock (brefeldin A solution (1,000x) (Invitrogen, 00-4506-51, China) and monensin solution (1,000x) (Invitrogen, 00-4505-51, China)) was added into the cell suspension and cultured for 4 hours at incubator. After centrifugation and washing, the suspension was added into fixing agent (fixation/permeabilization concentrate (Invitrogen, 00-5123-43, China): fixation/Permeabilization diluent (Invitrogen, 00 − 5223 − 56, China) = 1 : 3) and incubated for 1 hour at 4°C in the dark. The cells were punched using Permeabilization Buffer (Invitrogen, 00-8333-56, China). Following centrifugation and washing, PE anti-human IFN-*γ* (BioLegend, 506506, USA) was added into the cell suspension overnight at 4°C. Flow cytometry readings were performed on the FACS.

### 2.9. Western Blot

Total proteins were isolated using RIPA buffer (Solarbio, Beijing, China) containing protease and phosphatase inhibitors and stored at -20°C. The protein concentration was quantified by BCA Protein Assay Kit (Beyotime, China). Approximately 20 *μ*g of total proteins was separated by standard SDS-PAGE (10% or 12.5%) and transferred onto PVDF membranes by wet transfer. After blockage with NcmBlot Blocking Buffer (NCM Biotech, China), the PVDF membranes were hatched with primary antibodies p-PI3k (1 : 1,000, Cell Signaling Technology, USA, 4228T), PI3K (1 : 1,000, Cell Signaling Technology, USA, 4249T), p-ERK1/2 (1 : 1,000, Cell Signaling Technology, USA, 4370T), ERK1/2 (1 : 1,000, Cell Signaling Technology, USA, 4695T), MEK1/2 (1 : 500, Wanleibio, China, WL03328), GAPDH (1 : 1,000, ZEN-BIOSCIENCE, China, 380626), and NKG2D (KLRK1) (1 : 1,000, ZEN-BIOSCIENCE, China, 863091), at 4°C overnight. Next day, after washing, the PVDF membranes were incubated with corresponding secondary antibodies for 1 h at room temperature. Later, the membranes were washed again and detected using super ECL (SORFA, China) and the ChemiDoc MP Imaging System (Bio-Rad Laboratories, Hercules, CA, USA). The relative protein expression was calculated by the Image Lab software compared to internal control.

### 2.10. Statistical Analysis

All statistical analyses were performed using the GraphPad Prism 7 software and R software. Student's *t*-test and Mann–Whitney *U* test were used to compare the mean between the two groups. One-way analysis of variance (ANOVA) was performed in multiple groups. Kaplan–Meier curves were plotted using the R package “survival,” and differences between subgroups were compared by the log-rank test. All data were presented as mean ± standard deviation (SD). Statistical significance was described as follows: #*p* > 0.05, not significant; ∗*p* ≤ 0.05; ∗∗*p* ≤ 0.01; ∗∗∗*p* ≤ 0.001; and ∗∗∗∗*p* ≤ 0.0001.

## 3. Results

### 3.1. The Immune Infiltration Levels in WT

**Figure 1:**
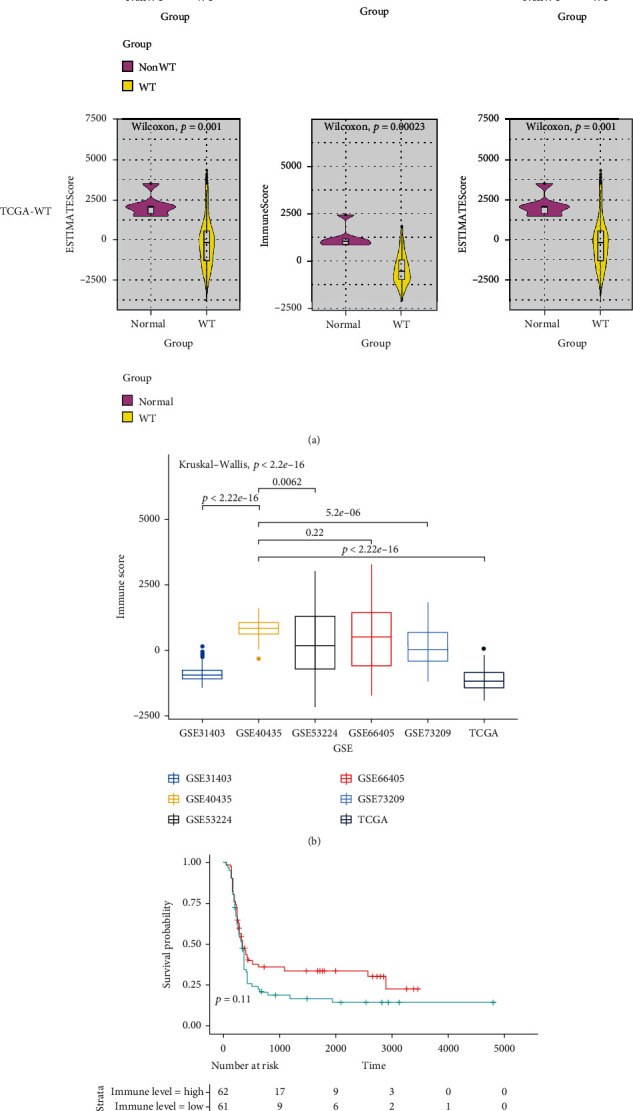
Bioinformatics analysis of infiltrating immune cells and stromal cells in WT samples. Compared the ESTIMATE algorithm results of immune cells, stromal cells, and estimate scores in WT sample and adjacent kidney sample by Wilcoxon test, respectively (a). Compared the immune scores of WT sample with the adult ccRCC (b). Kaplan-Meier survival analysis of the prognosis between high and low immune scores group (c).

Immune infiltration in tumor microenvironment (TME) plays a crucial role which is closely associated with the clinic outcome [13]. With the development of bioinformatics, the ESTIMATE algorithm provides a reliable method in evaluating immune and stromal cells in the TME [14]. In this study, an overview of ESTIMATE algorithm is shown in Figure 1(a). The results showed that the immune scores in WT were significantly lower than the non-WT in both TCGA and GSE-Merge data (Figure 1(a)). Meanwhile, in order to further explore the immune level in WT, we compared the WT samples with the adult ccRCC samples. The results showed that the immune scores in WT were lower in WT which compared with ccRCC which may be represented that the immune infiltration was low level in WT (Figure 1(b)). Interestingly, although immunity infiltration scores were low in WT samples, we found that different scores were correlated with the event-free survival (EFS) (Figure 1(c)). Hence, our results indicated that immune infiltration levels may cause the poor outcomes of WT.

### 3.2. NK Cell-Mediated Cytotoxicity Was Associated with the Immune Infiltration

**Figure 2:**
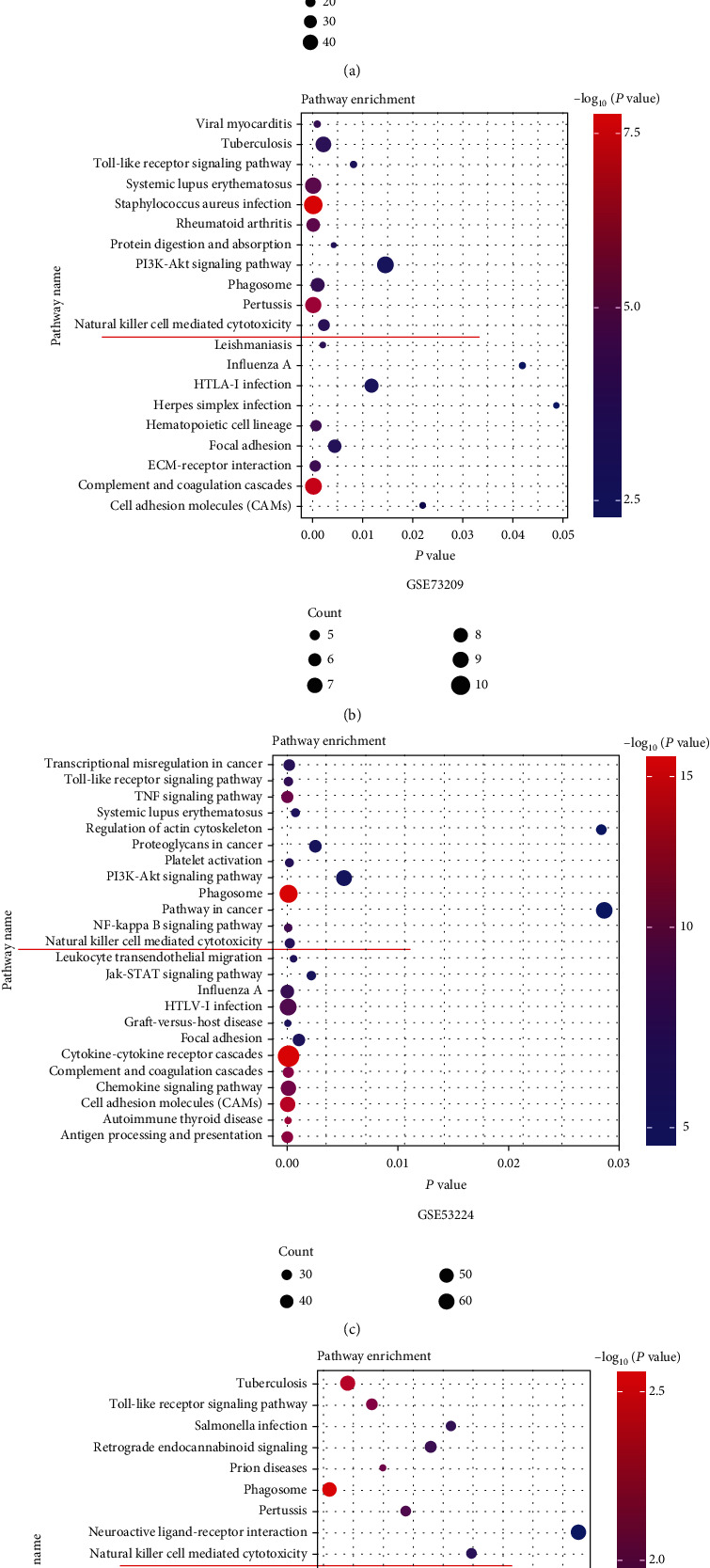
DAVID enrichment analysis of the differentially expression genes between high immune scores and low immune scores in GSE66405, GSE73209, GSE53224, and TCGA-WT datasets, respectively. Red line indicated that four datasets (signaling enrichment pathway name) were natural killer cell-mediated cytotoxicity.

**Figure 3:**
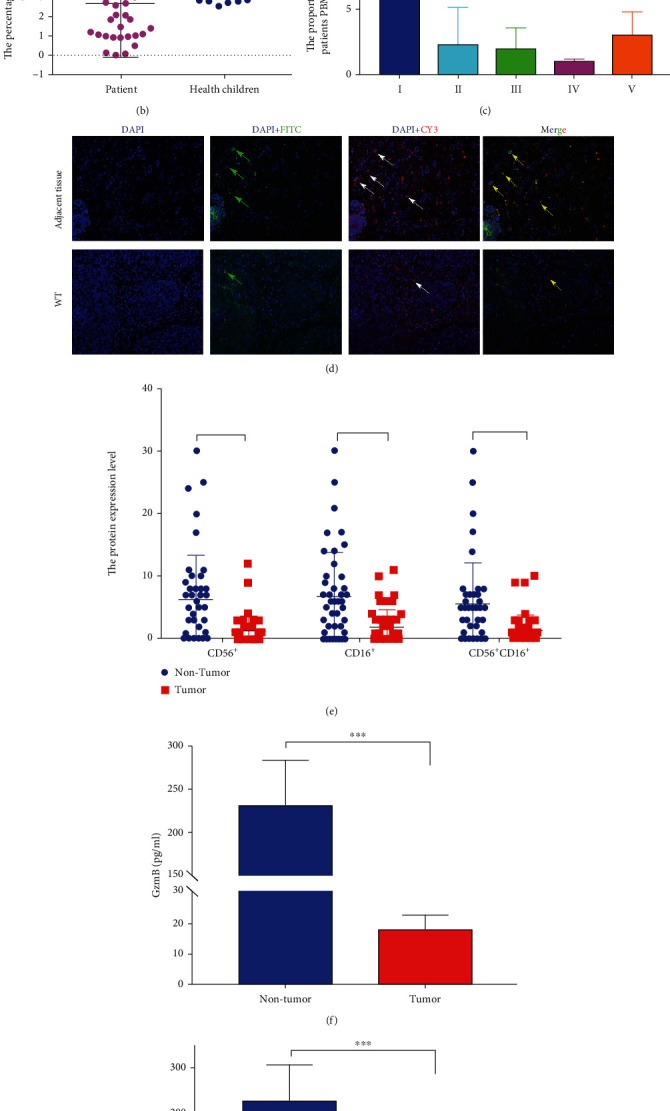
The NK cells were detected in WT clinic sample. The proportion of CD3^−^CD16^+^CD56^+^ NK in PBMCs were analyzed in WT patients (*n* = 30) and normal children (*n* = 30) by FACS (a, b). The proportion of CD3^−^CD16^+^CD56^+^ NK in WT patient PBMCs with different stage (c). The CD16^+^, CD56^+^ and CD16^+^, and CD56^+^ NK cells were detected in paired WT samples (*n* = 30) by immunofluorescence staining (d, e). Cytotoxic cytokines were detected by ELISA in paired WT samples (*n* = 30). Results are shown as mean ± SD. For comparisons, Student's *t*-test was performed in two group, or one-way analysis of variance (ANOVA) was performed in multiple groups. ^∗^*p* < 0.05, ^∗∗^*p* < 0.01, and ^∗∗∗^*p* < 0.001.

We have found abnormal immune infiltration in WT in the above result; however, the specific mechanism is not clear. In our study, immune-related DEGs were identified by comparing the RNA expression of WT datasets with high and low immune scores. We employed the DAVID to analyze the DEGs in four WT datasets, respectively. The detail is shown in Figure 2, and the GO enrichment analysis showed that NK cell-mediated cytotoxicity was significantly different among different immune score subgroups (Figure 2). Noteworthy, in most of tumor, NK cells play a key role in tumorigenesis with cytotoxic function [15]. In order to further explore the specific mechanism of NK cells in WT sample, the proportion of CD3^−^CD16^+^CD56^+^ NK cells in WT patients PBMC were analyzed by FACS. Although few of the proportion of CD3^−^CD16^+^CD56^+^ NK cells in patients PBMC were increased, most of that were significantly lower than normal children (Figures 3(a) and 3(b)). Moreover, compared to stage I patients, the CD3^−^CD16^+^CD56^+^ NK cells in stage II, III, and IV patients were significantly decreased (Figure 3(c)). Meanwhile, we detected the CD16^+^, CD56^+^, and CD16^+^CD56^+^ NK cells in paired WT samples by immunofluorescence staining. We found that the CD16^+^, CD56^+^, and CD16^+^CD56^+^ NK cells were significantly low expression in WT tissue (Figures 3(d) and 3(e)). As reported, NK cells respond to tumor cells by producing cytotoxicity cytokines, including INF-*γ*, PRF, and GZMB which directly targeted tumor cells to exerted their functions [16]. In order to explore the cytotoxicity of NK cells in WT, we found that the expression levels of GZMB, IFN-*γ*, and PRF1 were significantly decreased in WT tissue which compared with adjacent tissue (Figures 3(f)–3(h)). Taken together, our results indicated that low expression level and function of NK cells in WT may be a key factor that caused the development of WT.

### 3.3. Low Expression of NKG2D in WT

**Figure 4:**
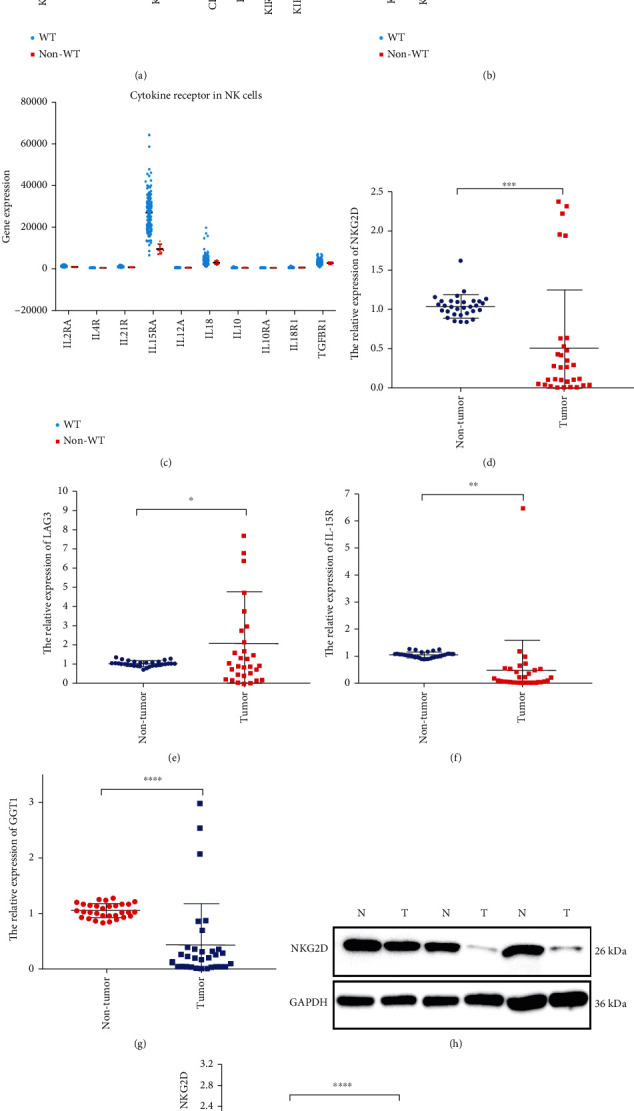
The receptor of NK cell analysis in TCGA dataset and paired WT samples. The gene expression level of activating receptor, inhibitory receptor, and cytokine receptor in NK cells of WT and adjacent kidney in TCGA-WT datasets (a–c). qRT-PCR analyzes the gene expression level of the NKG2D, LAG3 IL-15R, and GGT1 in paired WT sample, respectively (d–g). Quantification analysis of NKG2D protein expression level was performed by Western blot in paired WT sample (h, i). N and T represent nontumor adjacent kidney and WT, respectively. Results are shown as mean ± SD. For comparisons, Student's *t*-test was performed in two group, or one-way analysis of variance (ANOVA) was performed in multiple groups. ^∗^*p* < 0.05, ^∗∗^*p* < 0.01, ^∗∗∗^*p* < 0.001, and ^∗∗∗∗^*p* < 0.0001.

Normally, NK cells stay in a balance state and can be activated after infection, pathogen, or tumor invasion. The activation of NK cells depended on the surface receptor. In order to find the activation mechanism of NK cells in WT tissue, we further analyzed three types of receptor gene expression including activating, inhibitory, and cytokine receptors in TCGA datasets [17]. The results showed that NKG2D (KLRK1), CD224 (GGT1), CD226 (DNAM1), and IL-15RA were significantly low expression in WT tissue which compared with nontumor tissue (Figures 4(a)–4(c)). On the contrary, LAG3 was significantly high expression in WT tissue (Figures 4(a)–4(c)). We further detected the NKG2D (KLRK1), CD224 (GGT1), LAG3, and IL-15RA gene expression level in our paired WT samples. The results were consistent to TCGA dataset results (Figures 4(d)–4(g)). As previous studies showed, NKG2D plays an important role in NK cell activation [18]. The protein expression level of NKG2D was detected on paired WT samples. Compared with nontumor tissue, the expression level of NKG2D was significantly low in WT tissue (Figures 4(h) and 4(i)). These findings indicated that the abnormal activation of NK cell may be related to the low expression of NKG2D.

### 3.4. IL-2 and IL-15 Enhance the Cytotoxicity of WT Patient-Derived NK Cells

**Figure 5:**
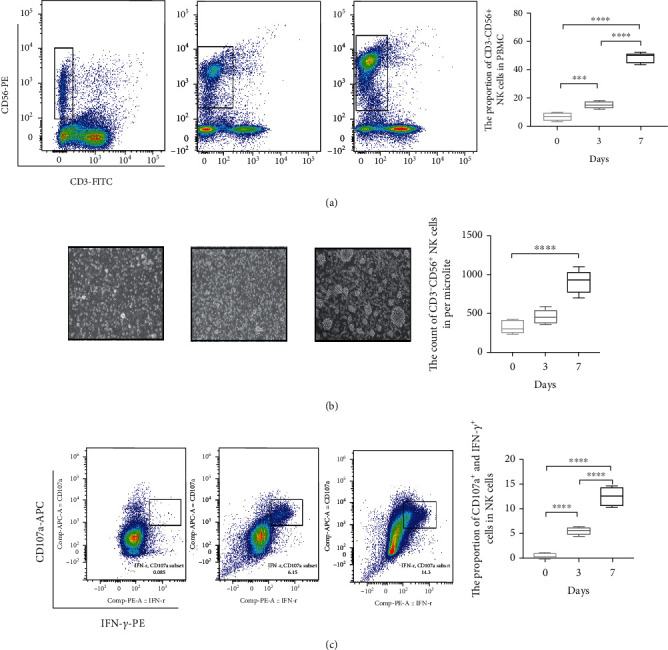
IL-2 and IL-15 enhance the quantity and cytotoxicity of patient autologous NK cells. Unculture and IL-2 and IL-15 costimulation culture 3 days and 7 days patient autologous CD3^−^CD56^+^ NK cell in PBMC detected by FACS (a). The statistical results of CD3^−^CD56^+^ NK cell in PBMC (a). Unculture and IL-2 and IL-15 costimulation culture 3 days and 7days CD3^−^CD56^+^ NK cell morphology observed under a microscope, respectively (b). The statistical results of CD3^−^CD56^+^ NK cell quantity in per microliter (b). Cytotoxic cytokine (CD107a and IFN-R) expression level of NK cells was detected by FACS (c). The proportion of CD107a + IFN-R NK cells (c). For comparisons, Student's *t*-test was performed in two group, or one-way analysis of variance (ANOVA) was performed in multiple groups. ^∗∗∗^*p* < 0.001 and ^∗∗∗∗^*p* < 0.0001.

Due to the upon results, WT patient-derived NK cells have a lower cytotoxicity which may cause the development of tumor. In order to explore the potential immune treatment strategy, cytokines could be beneficial in enhancing the antitumor immune activity mediated by NK cells [19]. In contrast to interleukin- (IL-) 2 alone, a stimulation of patient-derived NK cells with IL-15 plus IL-2 resulted in increasing the cytotoxicity and cytokine production in vitro and in vivo [20]. Our result showed that the proportion of CD3^−^CD56^+^ NK cells were significantly increased after 7 days of culture (Figure 5(a)). The morphology of CD3^−^CD56^+^ NK cells was better under microscope, and the count of CD3^−^CD56^+^ NK cells was significantly increased (Figure 5(b)). Importantly, the CD107a and IFN-*γ* of CD3^−^CD56^+^ NK cells were significantly increased after 7 days of culture which compared with the 0 day (Figure 5(c)).

**Figure 6:**
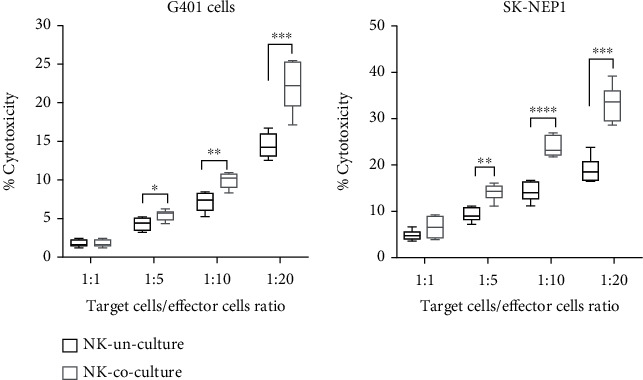
Cytotoxic activity of NK cells against WT cell lines (G401 and SK-NEP-1). Labeled G401 and SK-NEP-1 cells were incubated with NK cells derived with cytokines. Results are expressed as mean (SEM) percent of cytotoxicity at various E:T ratios. For comparisons, one-way analysis of variance (ANOVA) was performed in multiple groups. ^∗∗∗^*p* < 0.001 and ^∗∗∗∗^*p* < 0.0001.

To evaluate the tumor toxic effects of uncultured and cytokine (IL-2 and IL-15) coculture NK cells, we have chosen two types of tumor cell lines including human Wilms' tumor (G401 and SK-NEP-1) and as the targets in antitumor assay. After coculture with IL-15 and IL-2 for 7 days, the cell viabilities were measured for each type of tumor based on LDH method. The results indicated that killing effect of coculture NK cells on WT cell lines was significantly increased which compared with NK cells unculture when the effect-target ratio was 5 : 1, 10 : 1, and 20 : 1, respectively (Figure 6). Therefore, our results indicated that IL-2 and IL-15 could effectively enhance the number and function of WT patient-derived NK cells.

### 3.5. NK Cell Activation through MAPK Signaling Pathway

**Figure 7:**
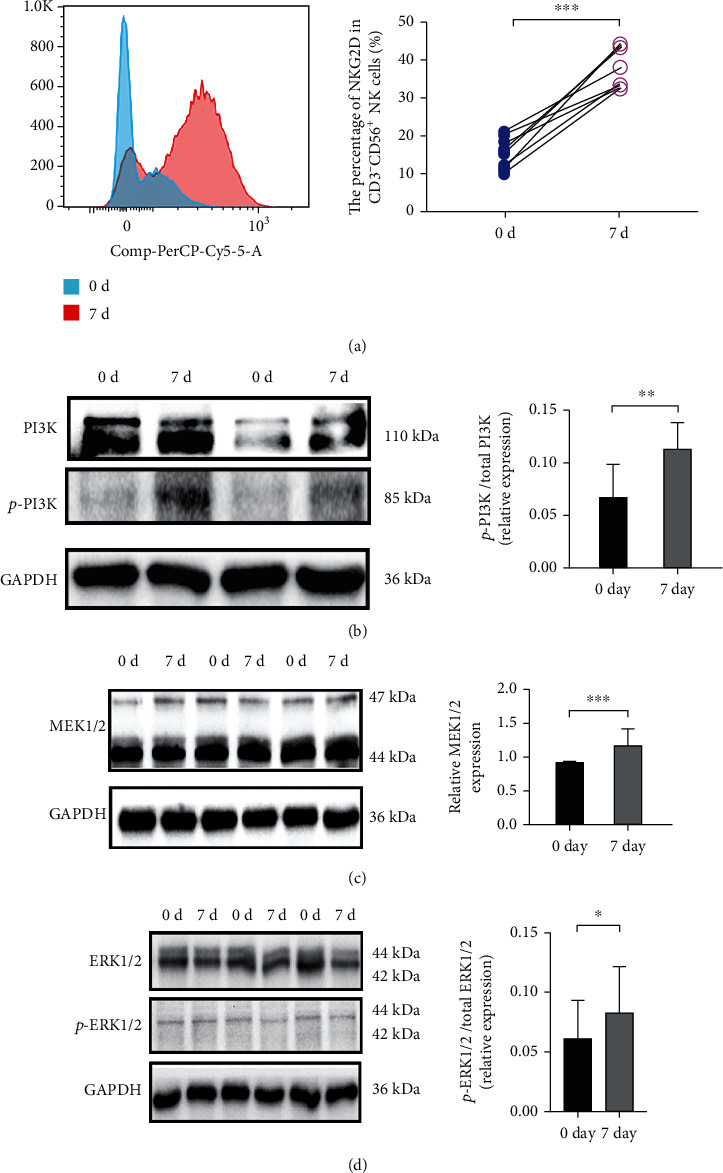
The protein expression level of IL-2 and IL-15 costimulation culture 0 and 7 days. The FACS analysis NKG2D expression percentage of NK cells (a). Western blot analysis of p-PI3K/PI3K, MEK, and p-ERK/ERK protein expression levels of the uncultured NK cells compared with the IL-2 and IL-15 costimulation culture 7 days NK cells. For comparison, Student's *t*-test was performed. ^∗∗^*p* < 0.01, ^∗∗∗^*p* < 0.001, and ^∗∗∗∗^*p* < 0.0001.

**Figure 8:**
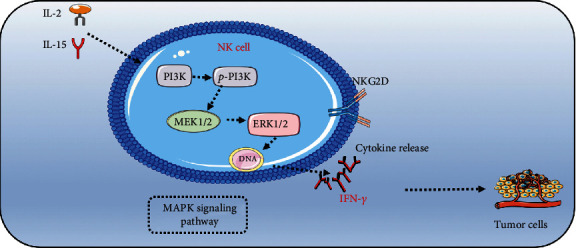
IL-2 and IL-15 enhance NK cell-mediated cytotoxicity through MAPK signaling.

As the reported on KEGG (map04650), the NK cell-mediated cytotoxicity involved in MAPK signaling pathway activation. In order to explore the specific mechanism of IL-2 and IL-15 in activation of WT patient-derived NK cells, we employed the FACS and Western blot (WB) on analysis of related protein expressions. The FACS results showed that the activation receptor (NKG2D) in NK cells was significantly increased in the cocultured group (Figure 7(a)). Meanwhile, our results showed that phosphorylation protein ratio of PI3K and ERK1/2 was significantly increased in the cocultured group which compared with the uncultured group (Figures 7(b) and 7(c)). Similarly, the protein level of MEK1/2 was significantly increased in the cocultured group (Figure 7(d)). Taken together, our results indicated that IL-2 and IL-15 could effectively enhanced the cytotoxicity of patient-derived NK cells to kill WT cells which may enhance the expression of NKG2D and activation of MAPK signaling pathway (Figure 8).

## 4. Discussion

Tumor-infiltrating lymphocytes (TILs) and cytotoxic cytokines play an important role in immune surveillance. Normally, activation of the immune system and proliferation of immune cells inhibit the development and metastatic of tumor. However, low level of TILs was associated with poor outcome in various tumors including ccRCC, liver cancer, and lung cancer. Hence, a comprehensive analysis of TILs in WT is meaningful to explore the potential immunotherapy strategy. In this study, we employed the ESTIMATE to evaluate multidataset gene sequencing results found that the immune scores in WT were significantly lower than adjacent non-WT kidney. Furthermore, compared with adults ccRCC, the immune scores in WT are still at a low level. Our finding was similar to the recently systematic review that indicated the presence of an immunosuppressive microenvironment in Wilms' tumor [21]. Importantly, high level of TILs was usually regarded as a beneficial factor for prognosis. However, unfortunately, high clinic stage of WT usually lacks a high number of macrophages, CD3^+^ lymphocytes, and CD8^+^ T cell infiltration [22–24]. In this study, although there was no significantly statistical difference, the low immune scores were associated with a poor EFS in WT. Immune score was related to prognosis, but not all immune-specific genes were prognostic factors. We took the intersection of high and low immunity to obtain DEGs in different datasets. GO analysis showed that multi-immune-related signaling pathway different may cause in low/high immune scores. Interestingly, a common pathway, NK cell-mediated cytotoxicity, was abnormal in four datasets. NK cell-mediated cytotoxicity is a key component of TILs on antitumor progression. One of the possible immunotherapeutic approaches is based on the antibody-dependent cellular cytotoxicity (ADCC), which is mediated by NK cells. ADCC of NK cells is including secretion of the cytotoxic granules (GZMB and PRF1), TNF-mediated signaling, and proinflammatory cytokine release (IFN-*γ*) [25, 26]. Here, we observe that the PRF1, GzmB, and IFN-*γ* in WT sample were significantly lower than adjacent nonkidney.

Limited availability of NK cell antitumor effect is mainly due to its quantity and function. In peripheral blood, the proportion of NK cell is usually 5-15%. In this study, we compared the proportion of NK cells in WT patients with normal children. We found that part of WT patient NK cells was increased, however, most of patients with a low proportion of NK cells. Meanwhile, the proportion of NK cells in WT patients with stage I were significantly higher than II-IV stages. We further explored the NK cells in paired WT sample and found that the number of CD16^+^CD56^+^ NK cells in WT sample was lower than adjacent kidney. Overall, our results indicated that the less quantity and cytotoxicity dysfunction of WT patients NK cells may cause the immune surveillance disorder in WT. As the previous reported, the expression of activating and inhibitory receptors on NK cell surface plays a key role on NK cell function [27]. The activating receptors and inhibitory receptors in some tumors were systematically analyzed by Martinović et al. [28]. The results showed that downregulated NKG2D, NKp46, and DNAM-1 receptors associated with impaired NK cell effector function are important biomarkers of advanced disease with a poor prognosis in melanoma patients [28]. Meanwhile, activating receptors directly interact with the overexpressed/de novo expressed ligands on transformed cells and provide NK cells with the ability to recognize and kill tumor cells regardless of their human leukocyte antigen- (HLA-) I expression. In this study, we found that the relative expression level of NK cells receptors (LAG3) in WT was lower than nontumor adjacent. However, the relative expression level of NK cell receptor (NKG2D, IL-15R, and GGT1) in WT was higher than nontumor adjacent. Meanwhile, NKG2D, via binding to NKG2D ligands (NKG2DLs), plays an important role in the immune response, including immune surveillance, antimicrobial immune response, and antitumor effects [29]. Moreover, some reports demonstrated that malignancy cells could escape immune surveillance in NKG2D-deficient mice [30]. In this study, we found that the protein expression level of NKG2D in WT tissue NK cells was significantly low than the adjacent nontumor kidney NK cells. The results indicated that low expression NKG2D in WT may cause the NK cell-mediated cytotoxicity disorder.

Cytokine-induced killer cells showed potent cytotoxicity against a variety of tumor cells [31]. Compared the IL-2 alone stimulation of NK cells, IL-1, IL-7, IL-15, and IL-12 have been employed in combination with IL-2 that has more activity [32]. Wang et al. demonstrated that CIK_IL-15_ exhibits enhanced proliferation capacity than CIK_IL-2_, whereas CIK_IL-2_ showed more efficient cytotoxic effect against tumor cells than CIK_IL-15_ [33].

In order to explore the potential of immunotherapy strategy on WT, cytokine therapy-induced NK cell functions are likely to affect the clinical course of several human malignancies, including neuroblastoma, gastrointestinal sarcoma, and kidney and lung carcinoma [34, 35]. In this study, WT patient autologous PBMCs were stimulated by IL-2 combination with IL-15. This result indicated that IL-2 and IL-15 could effectively enhance the proportion and count of NK cells in PBMCs. Meanwhile, LDH assay demonstrated that the capacity of against WT cell lines was significantly increased by IL-2 and IL-15 cultured NK cells. The expanded cells are functional as both IFN-*γ* and CD107a expressions on NK cells are increased. Furthermore, NK cell-mediated cytotoxicity involved that both ITAM-dependent signaling and DAP-10-dependent signaling seem to converge on a common NK cell cytotoxicity pathway. This common pathway involves the activation of Rac, which sequentially leads to activation of the mitogen-activated protein kinase (MAPK) extracellular signal-regulated kinase (ERK) by way of p21-activated kinase (PAK) and mitogen-activated or extracellular signal-regulated protein kinase kinase (MEK) [36]. In this study, we found that IL-2 and IL-15 stimulations of NK cells could enhance the key protein expression level of MAPK which involved in PI3K, MEK, and ERK. Meanwhile, IL-2 and IL-15 stimulations of NK cell could effectively enhance the NKG2D positive proportion.

## 5. Conclusion

In this study, we found that the cytotoxicity abnormal of NK cells in WT is associated with the poor outcomes in WT. Moreover, low infiltrating number and cytotoxicity dysfunction of NK cells in tumor microenvironment may cause the development of WT. An effective strategy is to expand and activate WT patient-derived NK cells in vitro via IL-2 and IL-15. Meanwhile, IL-2 and IL-15 could enhance the cytotoxicity of NK cells on inhibiting the growth of WT cells. The specific mechanism is through NKG2D-mediated MAPK signaling pathway.

## Data Availability

The data of this study are available from the corresponding author upon request.
